# Prognostic impact of tumor‑associated stroma in triple-negative breast cancer

**DOI:** 10.1007/s12282-024-01661-8

**Published:** 2024-12-18

**Authors:** Akinari Kakumoto, Tsengelmaa Jamiyan, Ai Koyanagi, Hajime Kuroda, Rin Yamaguchi, Hitoshi Tsuda, Akira Hirano, Shunichi Shiozawa

**Affiliations:** 1 Department of Diagnostic Pathology, Tokyo Women’s Medical University Adachi Medical Center, 4-33-1 Kohoku, Adachi-ku, Tokyo 123-8558 Japan; 2 Department of Pathology and Forensic Medicine, Mongolian National University of Medical Sciences, Ulan Bator, 14210 Mongolia; 3 Department of Diagnostic Pathology, Nagasaki University Hospital, 1-7-1 Sakamoto, Nagasaki City, Nagasaki 852-8521 Japan; 4 Department of Pathology II, National Defense Medical College, 3-2 Namiki, Tokorozawa, Saitama 359-8513 Japan; 5 Department of Breast Surgery, Tokyo Women’s Medical University Adachi Medical Center, 4-33-1 Kohoku, Adachi-ku, Tokyo 123-8558 Japan; 6 Department of Surgery, Tokyo Women’s Medical University Adachi Medical Center, 4-33-1 Kohoku, Adachi-ku, Tokyo 123-8558 Japan

**Keywords:** Tumor-associated stroma, Triple-negative breast cancer, Tumor-infiltrating lymphocytes

## Abstract

**Aim:**

To establish the histological categorization of tumor‑associated stroma (TAS) that reflects the biological behavior of triple-negative breast cancer (TNBC).

**Methods and results:**

One-hundred-and-twenty surgically resected cases of TNBC were examined. We histologically categorized the TAS in the invasive frontal region into two groups: mature stroma (MS) and immature stroma (IS). The designation of IS was applied for tumors in which the largest myxoid stroma filled a high-power magnification field. When there were no myxoid stroma that meet the criteria for IS, TAS was categorized as MS. The tumors with type MS were observed in 103 (85.8%) of patients, whereas 17 (14.2%) of patients had tumors with IS. In total, 72 out of 120 patients with TNBC exhibited high tumor-infiltrating lymphocytes (TILs) representing 60% of the cohort. The incidences of high TILs were 66% (68 out of 103) in the MS group but only 23.5% (4 of 17) in the IS group (*p* = 0.001). Progression-free survival (PFS) and overall survival (OS) curves were different between IS and MS groups (*p* < 0.001 each), and Cox multivariate regression analysis revealed that IS was an independent indicator for lower PFS and OS rates (*p* < 0.001; *p* = 0.008).

**Conclusion:**

Our findings suggest that TAS characteristics, particularly the distinction between IS and MS, play a significant role in the prognosis of TNBC. The presence of IS, associated with poor prognosis and low TILs, contrasts with the favorable outcomes observed in cases with MS. Understanding these TAS dynamics could aid in identifying patients with varying prognostic outcomes in TNBC, necessitating further research into the mechanisms behind these observations.

## Introduction

Gene expression profiling studies have divided invasive breast cancer into several major subtypes [1]. Of these, the so-called ‘triple-negative breast carcinoma’ (TNBC) marked by the absence of estrogen receptor (ER) and progesterone receptor (PgR) expression, as well as the lack of human epidermal growth factor receptor 2 (HER2) protein overexpression. This subtype is associated with an unfavorable prognosis [2, 3].

According to current clinical guidelines for breast cancer, the treatment decisions are typically guided by pathologic factors routinely assessed in clinical practice. Important factors influencing breast cancer prognosis and treatment decisions include primary tumor size, lymph node status, tumor grade, hormonal receptor status, and HER2 status [4]. However, for TNBC, the existing risk assessment factors may not consistently provide optimal patient stratification for treatment decisions. The tumor‑associated stroma (TAS) component surrounding cancer cells plays a significant role in tumor development and behavior across various organs, as highlighted in several studies discussing the influence of TAS in invasive breast cancers [5–15]. Despite evidence suggesting that TAS influences tumor behavior and could serve as a predictive factor, no prognostic marker with practical clinical application has been identified.

Traditionally, tumor microenvironment has been classified into two primary categories, i.e., tumor-infiltrating immune cells and TAS, each believed to play a crucial role in the advancement of cancer. First, there are tumor-infiltrating immune cells such as lymphocytes and macrophages, which are considered to represent the host immune response to cancer cells. Notably, medullary breast carcinoma and those displaying medullary features are characterized by significant infiltration of tumor-infiltrating lymphocytes (TILs) and macrophages. This subtype was associated with a more favorable prognosis compared to other high-grade cancers at the same stage [14]. Second, there is the category of TAS. However, there are conflicting reports on the relationship between the response of TAS and tumor invasion and metastasis. The histological studies have indicated that TAS is associated with cancer aggressiveness in several tumors such as colorectal cancer, breast cancer, and lung cancer [5–7]. Conversely, in vivo findings have supported the hypothesis that TAS might act as a protective mechanism against neoplasms [16, 17]. Based on these previous laboratory findings that underscore the critical role of TAS in determining tumor cell invasion and metastasis, we hypothesized that specific histological features in the TAS of TNBCs could closely correlate with cancer biology. Thus, we conducted the current study to assess the prognostic significance of TAS parameters in TNBC. Subsequently, we examined the correlation between these types of TAS and clinicopathological outcomes to establish the histological categorization of TAS that reflects the biological behavior of TNBC.

## Materials and methods

### Patients

The subjects were 120 patients with TNBC, who underwent surgical resection at Tokyo Women’s Medical University, Adachi Medical Center between 1999 and 2023. Patients’ clinical information was retrieved from institutional medical records. The clinical outcomes were also documented. For each case, all available hematoxylin and eosin (H&E)-stained whole-tissue sections were reviewed to confirm the diagnosis of mammary disease with no knowledge of prior histological results or clinical outcomes. The present study was approved by the Ethics Committees of Tokyo Women’s University (Tokyo, Japan; registration number 2023-0025).

### Immunohistochemistry (IHC)

IHC was performed using monoclonal antibodies against estrogen receptor (ER) (clone SP1, Novocastra (Leica), prediluted, nuclear), progesterone receptor (PgR) (clone 1E2, Novocastra (Leica), prediluted, nuclear), human epidermal growth factor receptor 2 (HER2) (clone 4B5, Roche (Ventana), prediluted, membranous). ER and PgR expression was estimated and > 1% nuclear-stained tumor cells were considered positive according to the American Society of Clinical Oncology and the College of American Pathologists (ASCO/CAP) guidelines [18]. HER2-negative status was confirmed as a staining score of 0/1 + . In cases with an IHC score of 2 + , fluorescence in situ hybridization (FISH) was used to determine the HER2 status, and it was considered negative when the ratio of HER2 to CEP17 was < 2.0. An IHC score of 3 + was classified as HER2-positive.

### TAS type classification

**Fig. 1:**
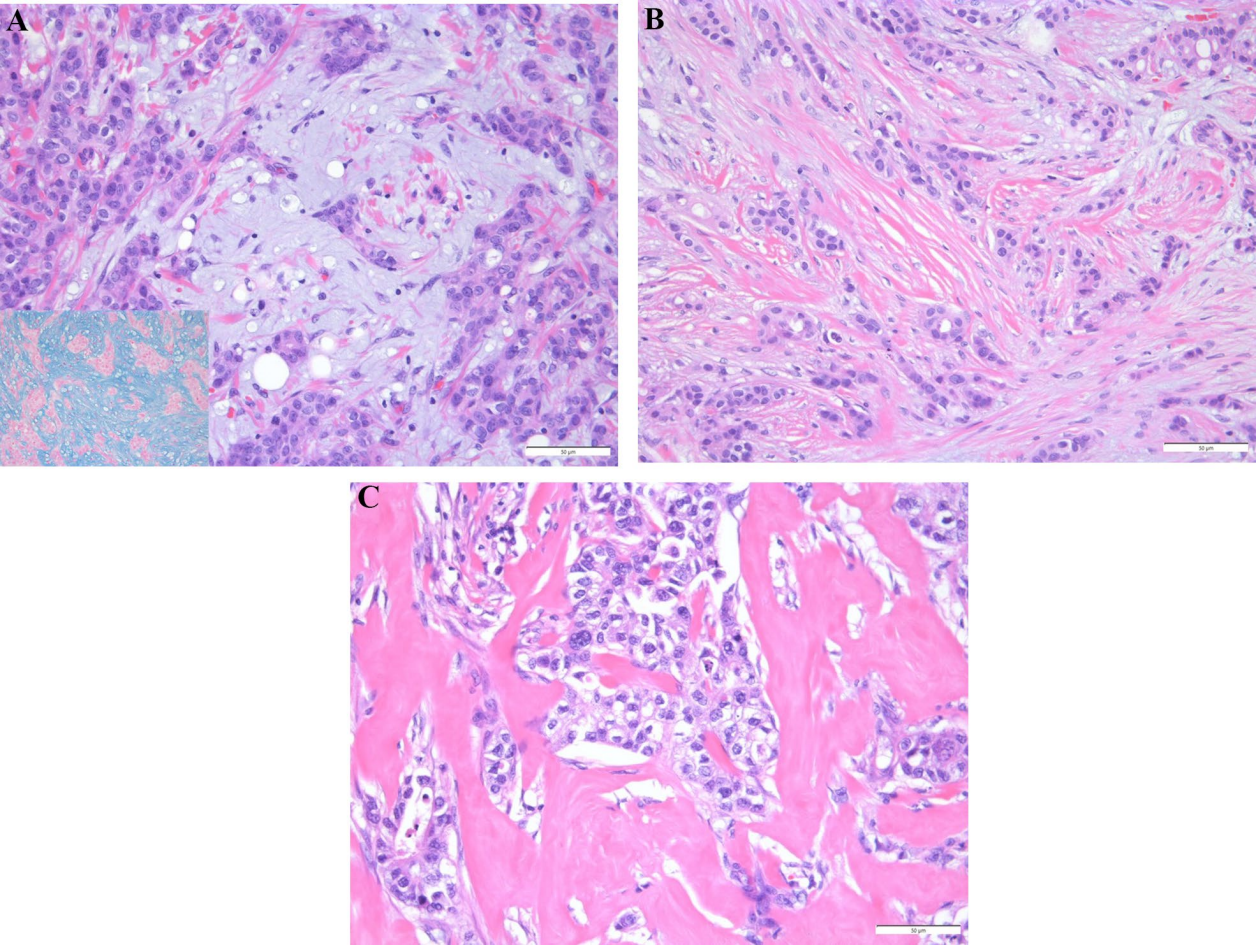
**A** Immature stroma (IS): myxoid stroma, an amorphous stromal substance comprising slightly basophilic extracellular matrix, appearing around the tumor nests. The document includes an inset image showing the Alcian blue staining. **B** Mature stroma (MS): fine and mature fibers with collagen fibers stratified into multilayers. **C** Mature stroma (MS): keloid-like collagen, such as hypocellular collagen with eosinophilic hyalinization, at the surrounding of the tumor

**Fig. 2:**
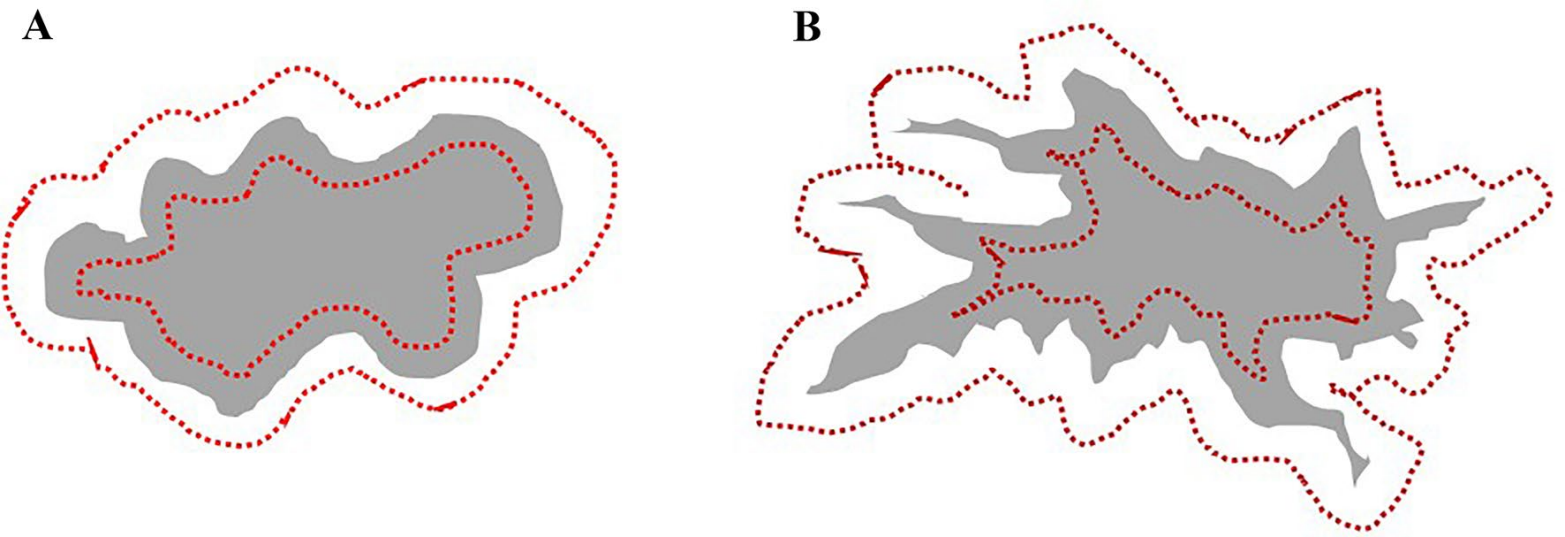
The evaluation of TAS was conducted in the extratumoral area outlined by the red line

Two pathologists (AK and HK), blinded to the clinical information, independently evaluated all histological sections. Any discrepancies in their assessments were resolved through consensus discussion and a final diagnosis was reached based on mutual agreement. The classification of TAS in this study was modified based on criteria outlined in a previous investigation [19, 20]. We histologically categorized the TAS in the invasive frontal region into two groups: mature stroma (MS), and immature stroma (IS). The flowchart for the process of categorizing TAS is as follows. H&E slides were first scanned at low-power magnification to identify neither myxoid stroma: an amorphous stromal substance comprising slightly basophilic extracellular matrix (Fig. 1A), or elongated collagen fiber stratification into multiple layers (Fig. 1B) nor a keloid-like collagen: hypocellular collagen with bright eosinophilic hyalinization (Fig. 1C) in the extratumoral area. Extratumoral area for solid type carcinoma (Fig. 2A) and infiltrative carcinoma such as scirrhous or invasive lobular (Fig. 2B) are shown as schematic diagrams. The designation of IS was applied for tumors in which the largest myxoid stroma filled a high-power magnification field of a 40 × objective lens completely. When there were no myxoid stroma that met the criteria for IS, TAS was categorized as MS. The evaluation was conducted using H&E staining. We used H&E-stained slides to distinguish between cancer-produced mucin and the stromal matrix. In these sections, mucin-producing cancer cells typically present pale blue mucinous material. In mucinous carcinoma, mucin concentrated within tumor cells or glandular lumens appears as pale blue. In contrast, myxoid stroma is characterized by scattered extracellular matrix, differing from the more concentrated appearance of tumor-produced mucin. When mucinous material is confined to tumor cells or glandular lumens, it is likely tumor-produced mucin. If mucin extends beyond the tumor cells, additional evaluation is required to determine whether it is leaked mucin from the tumor or a feature of the myxoid stroma itself. In cases where differentiation was difficult, Alcian blue (AB) staining was used for further evaluation (Fig. 1A). AB specifically stains for acidic groups, such as carboxyl or sulfate groups, which are present in the tumor-produced mucin or in the myxoid matrix within the stroma. Therefore, both the mucin and the stromal myxoid matrix, which mainly consist of O-glycans and proteoglycans respectively, are positive for AB staining. In contrast, the edematous stroma, which lacks the acidic group, is not AB positive. Thus, AB staining is useful in distinguishing between the edematous stroma and the myxoid matrix, even when they are difficult to distinguish by H&E staining.

### Evaluation of TILs

We assessed TILs on H&E-stained sections using the criteria proposed by the International Immuno-Oncology Biomarkers Working Group [21]. TILs were defined as mononuclear cells, including lymphocytes, within the TAS area, excluding necrosis, crush artifacts, regressive hyalinization, granulocytes, and other polymorphonuclear leukocytes (see Fig. 1). TILs levels were stratified as either high (≥ 30%) or low (< 30%), based on previously established cut-offs [22]. The quantification of TILs was performed in each case at × 400 magnification (× 40 objective). Two pathologists (AK and HK), blinded to patients’ clinical information, independently evaluated all sections, and the results were averaged.

### Statistical analysis

The relationships between TAS characteristics and clinicopathological variables were assessed using the chi-square test. Progression-free survival (PFS) was defined as the time from surgery to the occurrence of disease recurrence, including metastatic disease, and overall survival (OS) was calculated from the surgical date to the date of cancer-related death or the last follow-up. The survival curves were generated using the Kaplan–Meier method, and differences between groups were evaluated with the log-rank test. For both PFS and OS, hazard ratios (HRs) and 95% confidence intervals (CIs) were calculated using Cox proportional hazard models. A multivariate Cox regression analysis was performed to assess the independent effects of each variable, including those found to be statistically significant in the univariate analyses. The statistical significance was defined as a *p* value < 0.05 for all analyses, unless otherwise adjusted. All statistical analyses were conducted using IBM SPSS Statistics version 25 (IBM, Armonk, NY, United States).

## Results

The median age of breast cancer patients was 63 (range 30–91) years and 59.2% were older than 60 years. Tumors measuring ≤ 2 cm were observed in 69 (57.5%) patients. The majority of patients had a high histological grade, with 81 (67.5%) cases, and most had invasive ductal carcinoma (IDC), with 108 (90.0%) cases. The lymph node metastasis was observed in 34 (28.3%) patients. In total, 72 out of 120 patients with TNBC exhibited high TILs, representing 60% of the cohort. The tumors with type MS were observed in 103 (85.8%) of patients, whereas 17 (14.2%) of patients had tumors with IS. The incidence of high TILs was 66.0% (68 out of 103) in the MS group, compared to only 23.5% (4 out of 17) in the IS group (*p* = 0.001). There was a decrease in IS with high TILs, which accounted for 4 out of 17 (23.5%) cases. The median follow-up for the PFS and OS analysis was 79 (0–215) months. Univariate and multivariate Cox regression analyses of PFS and OS were performed using clinicopathological prognostic factors and TAS type (Table 1). Recurrence occurred in 22 (18.3%) and cancer-related death occurred in 18 (15.0%) out of 120 patients.

**Table 1:** Impacts of stromal type and tumor-infiltrating lymphocytes (TILs) on patient survival according to Cox univariate and multivariate analyses

Clinicopathological feature	PFS				OS			
	Univariate analysis		Multivariate analysis		Univariate analysis		Multivariate analysis	
	HR (95.0% CI)	*p* value*	HR (95.0% CI)	*p* value*	HR (95.0% CI)	*p* value*	HR (95.0% CI)	*p* value*
Age (< 60 vs. ≥ 60)	0.44 (0.18–1.03)	0.059			0.65 (0.25–1.64)	0.364		
Tumor size (2 cm vs. > 2 cm)	1.76 (0.76–4.08)	0.185			3.94 (1.40–11.0)	0.009	3.80 (1.33–10.8)	0.012
Histological grade (I, II vs. III)	0.81 (0.34–1.94)	0.650			1.26 (0.45–3.54)	0.657		
Histology (IDC vs. ILC, other types)	0.16 (0.005–5.10)	0.303			0.16 (0.004–7.56)	0.355		
Lymph node status (Absent vs present)	4.43 (1.89–10.40)	0.001	2.40 (0.93–6.22)	0.070	6.37 (2.38–17.01)	< 0.001	4.48 (1.61–12.4)	0.004
Stromal type (Mature vs immature)	12.5 (5.30–29.6)	< 0.001	8.27 (3.10–22.0)	< 0.001	4.86 (1.88–12.58)	0.001	3.82 (1.41–10.3)	0.008
TILs (Low vs. high)	0.22 (0.08–0.56)	0.002	0.65 (0.20–2.07	0.473	0.67 (0.25–1.80)	0.432		

*TNBC* triple-negative breast cancer, *DFS* disease-free survival, *OS* overall survival, *HR* hazard ratio, *CI* confidence interval, *IDC* invasive ductal carcinoma, *ILC* invasive lobular carcinoma, *TILs* tumor-infiltrating lymphocytes

*p value is significant

Tumors exhibiting IS characteristics demonstrated a higher correlation with lymph node metastasis compared to those tumors with MS features (*p* = 0.015). High TILs were observed much less in the TAS of tumors with IS compared with those with MS (*p* = 0.001). Further, the TAS pattern was not linked to the other parameters (age, tumor size, histological grade, histology) (Table 2).

**Table 2:** Clinicopathological features of triple-negative breast cancer (TNBC) and stromal type (*n *= 120)

Clinicopathological feature	Total no. of cases	Stromal type		
		Mature	Immature	*p* value*
Age (years)				
< 60	49	39	10	0.103
≥ 60	71	64	7	
Tumor size (cm)				
≤ 2	69	59	10	0.905
> 2	51	44	7	
Histological grade				
I & II	39	31	8	0.167
III	81	72	9	
Histology				
IDC	108	91	17	0.389
ILC	3	3	0	
Other types	9	9	0	
Lymph node status				
Absent	86	78	8	0.015
Present	34	25	9	
TILs				
Low (≤ 30%)	48	35	13	0.001
High (> 30%)	72	68	4	

*TNBC* triple-negative breast cancer, *IDC* invasive ductal carcinoma, *ILC* invasive lobular carcinoma, *TILs* Tumor-infiltrating lymphocytes

*p value is significant, χ^2^ test and *Fisher’s exact* test

Multivariate Cox regression analyses revealed that TAS type was an independent prognostic factor for PFS (HR = 8.27, 95% CI 3.1–22.5, *p* < 0.001). Moreover, tumor size, lymph node status, and TAS type emerged as independent prognostic factors for OS in multivariate analyses (HR = 3.80, 95% CI 1.3–10.8, *p* = 0.012; HR = 4.48, 95% CI 1.65–12.4, *p* = 0.004; HR = 3.82, 95% CI 1.4–10.3, *p* = 0.008).

### Impact of maturation of TAS on survival curve

**Fig. 3:**
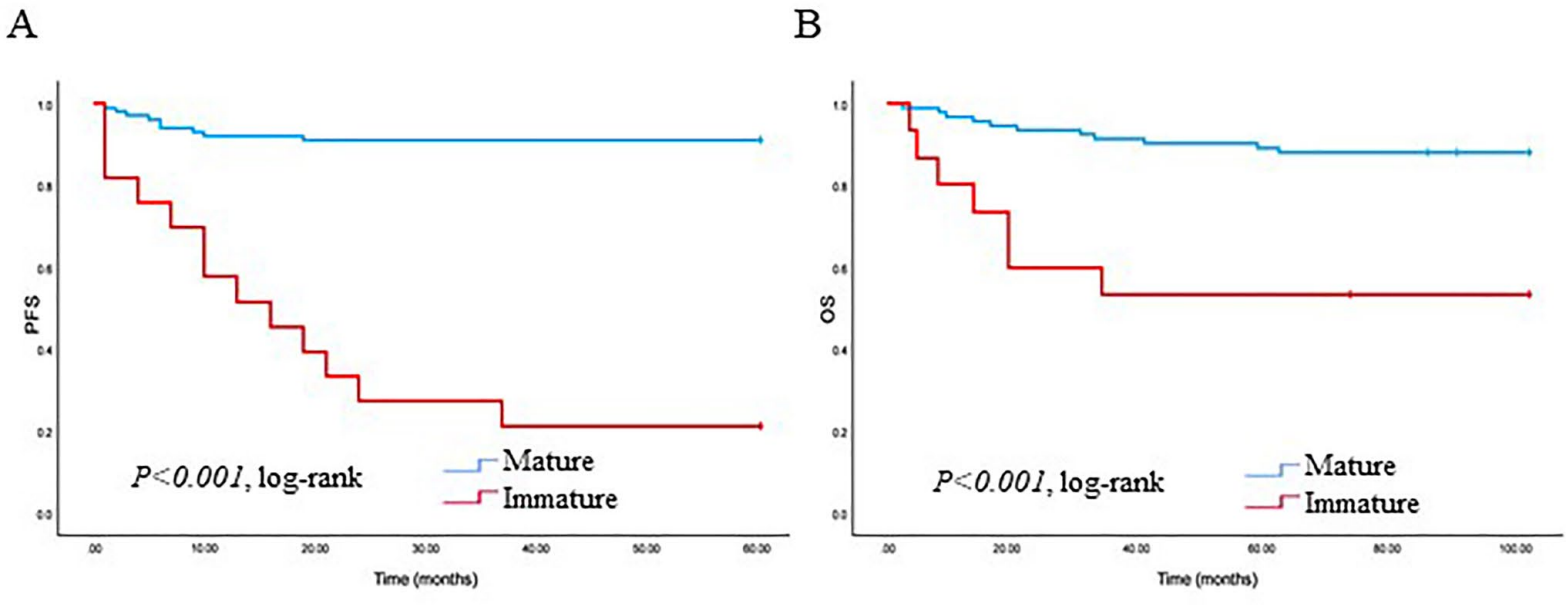
Kaplan–Meier plots showing progression-free survival (PFS) and overall survival (OS) in triple-negative breast cancer (TNBC) according to the type of tumor-associated stroma (TAS) (MS and IS). Log-rank tests were used to estimate P-values

**Fig. 4:**
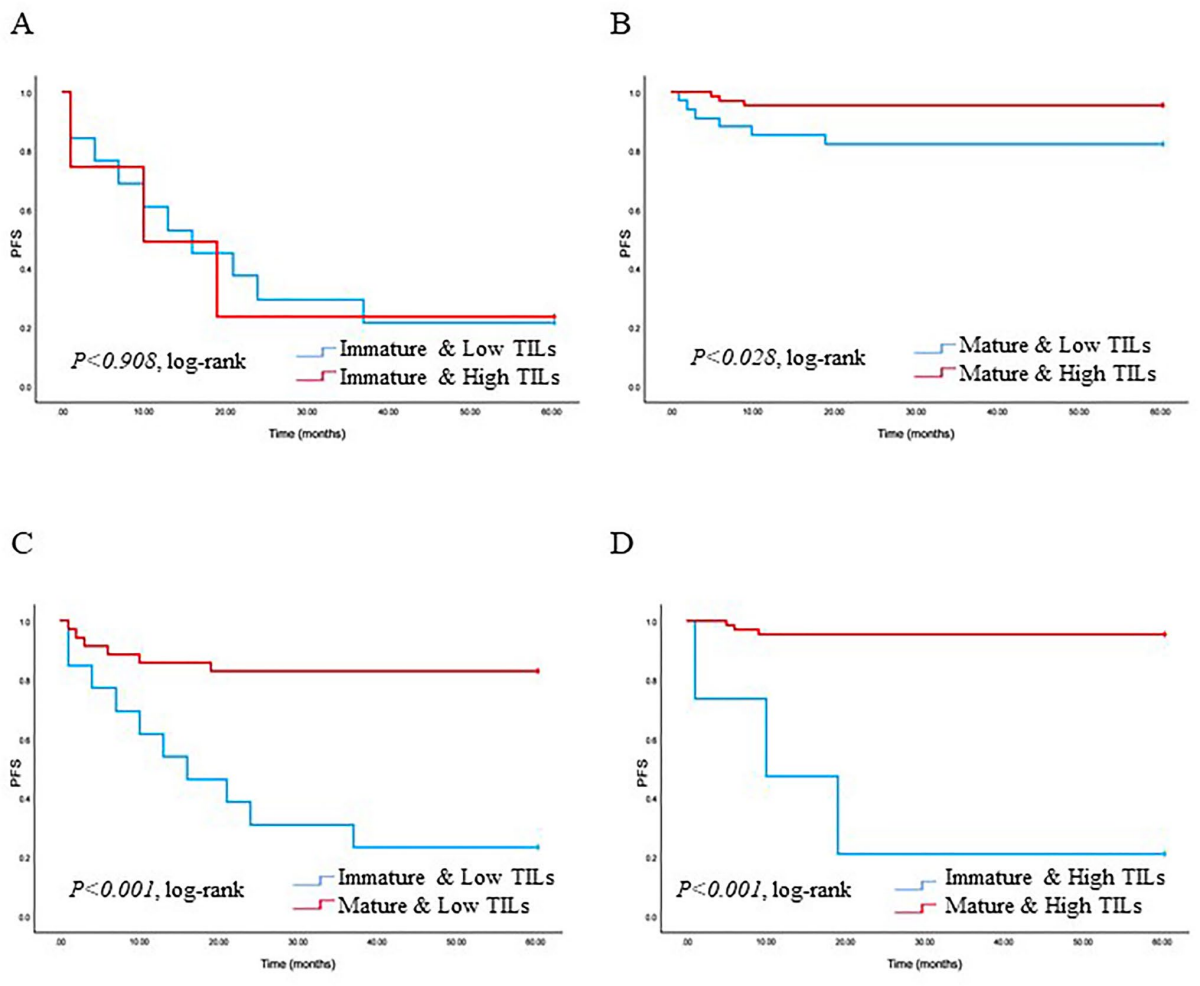
Kaplan–Meier plots showing PFS in TNBC according to TAS type and infiltration of tumor-infiltrating by lymphocytes (TILs). Log-rank tests were used to estimate *p* values. A. IS & low TILs (*n* = 13) vs IS & high TILs (*n* = 4), B. MS & low TILs (*n* = 35) vs MS & high TILs (*n* = 68), C. IS & low TILs (*n* = 13) vs MS & low TILs (*n* = 35), D. IS & high TILs (*n* = 4) vs MS & high TILs (*n* = 68), Graph shows *p* = log-rank test *p* values

**Fig. 5:**
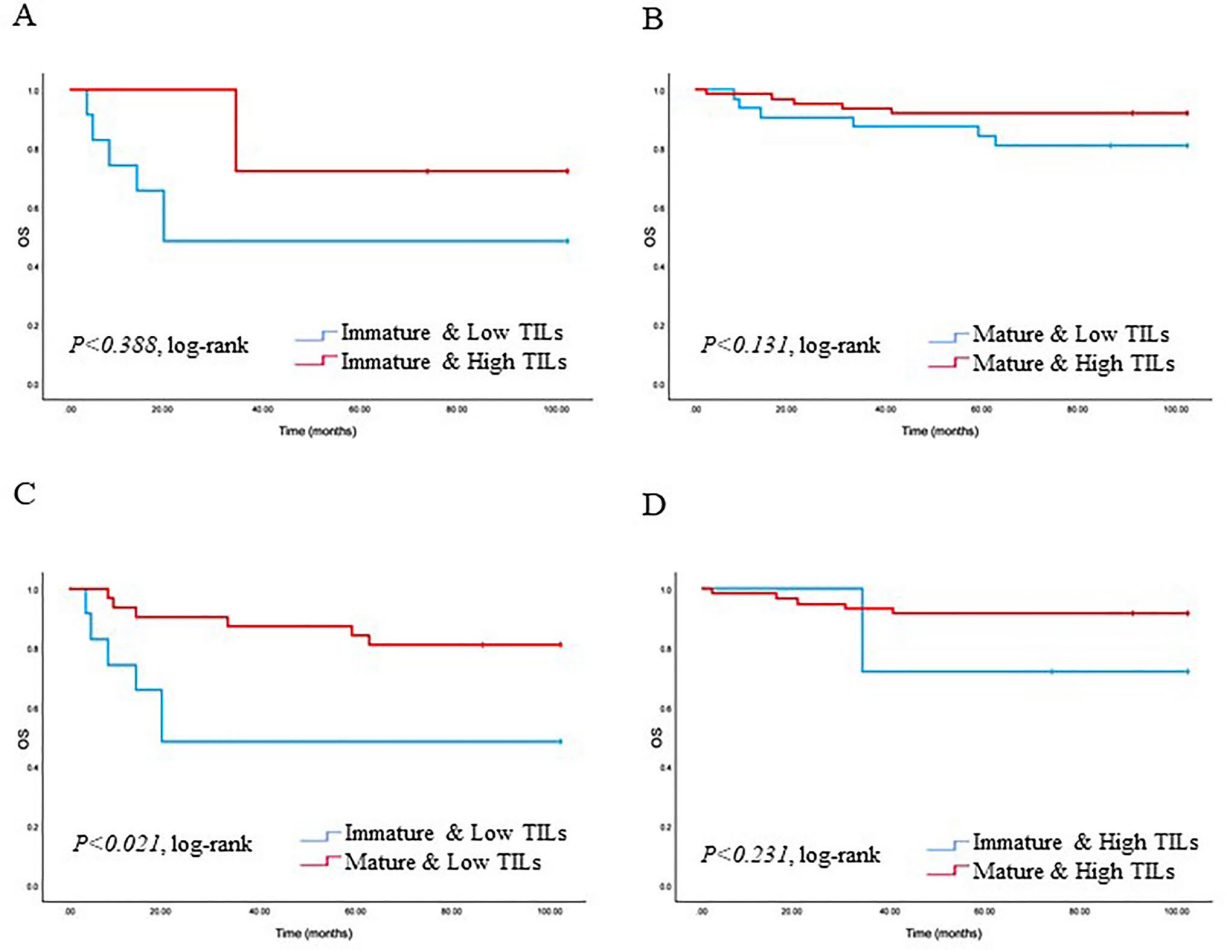
Kaplan–Meier plots showing OS in TNBC according to TAS type and infiltration of TILs. Log-rank tests were used to estimate *p* values. A. IS & low TILs (n = 13) vs IS & high TILs (*n* = 4), B. MS & low TILs (*n* = 35) vs MS & high TILs (*n* = 68), C. IS & low TILs (*n* = 13) vs MS & low TILs (*n* = 35), D. IS & high TILs (*n* = 4) vs MS & high TILs (*n* = 68), Graph shows *p* = log-rank test *p* values

We analyzed the correlation of TAS type with PFS and OS in patients with TNBC. The patients with tumors with MS had a good prognosis, while those with IS were correlated with poorer PFS and OS (*p* < 0.001 and *p* < 0.001) (Fig. 3). Examining the TAS types and TILs with PFS and OS is illustrated (Figs. 4 and 5). Tumors characterized by high TILs exhibited a more favorable PFS compared to those with tumors displaying low TILs in the MS (*p* = 0.028). Furthermore, low TILs in IS was associated with poorer PFS when compared to patients with a low TILs in MS (*p* < 0.001). Additionally, high TILs in IS was linked to poor PFS when contrasted with patients exhibiting high TILs in MS (*p* < 0.001). In contrast, there is no statistical significance between low TILs and high TILs in IS (*p* = 0.908). The patients with tumors displaying low TILs in IS exhibited a less favorable OS compared to those with tumors featuring low TIL infiltration in MS (*p* = 0.021). However, no statistical significance was observed between low TILs and high TILs in IS (*p* = 0.388), as well as MS with both low and high TILs (*p* = 0.131). Furthermore, there were no statistically significant differences noted among high TILs in both MS and IS (*p* = 0.231).

## Discussion

Our study showed that TNBCs exhibiting IS were found to be associated with an unfavorable prognosis in both PFS and OS (Fig. 3). Conversely, MS showed a correlation with a good prognosis.

It has been suggested that the TAS could represent an attempt by the host to prevent tumor cells, thereby exerting antagonistic biological forces. However, in respect of the relationship between the response of the TAS and tumor invasion and metastasis, there are some conflicting reports. In vivo findings supported the hypothesis that increased collagen synthesis in TAS might act as a protective mechanism against neoplasms. For instance, Barsky and Gopalakrishna observed that inhibiting the TAS response with L-3,4-dehydroproline led to an increase in spontaneous metastasis from experimental murine melanomas [16]. Similar to their findings, Nakanishi et al. identified an inverse correlation between the host’s TAS response and spontaneous lung metastasis in their study involving low and high metastatic murine lung carcinoma cell lines [17]. However, in histological studies, the TAS response does not consistently act in a way that suppresses the tumor. It is well known that in solid cancers such as colon cancer show myxomatous change in TAS [19]. Ueno et al. reported that IS was observed in 12% of their patients in colon cancer, which correlated with a poor prognosis [23]. Although only a few studies have focused on the IS in breast cancer, Zhai et al. recently reported that breast cancer with IS including all molecular subtypes showed a significantly poor prognosis [24]. Similar to our findings, Yanai et al. evaluated TAS in TNBC patients and reported that presence of IS (myxoid stroma) was significantly correlated with poor prognosis [25]. These findings advocate the hypothesis that some type of TAS response induce tumor aggressiveness.

Our findings demonstrate that, compared to IS, MS is significantly correlated with favorable pathological characteristics, such as lymph node status and prognosis. In multivariate analysis, TAS type emerged as an independent factor for both PFS and OS. This result indicates that TAS plays a complex role in cancer progression, possibly involving distinct mechanisms that have opposing effects on tumor behavior. Therefore, the TAS which classified into two types—IS and MS—each exerting different influences on the tumor microenvironment.

The TAS is composed of various elements, traditionally categorized into two main factors believed to contribute significantly to the progression of solid tumors like TNBC: the stroma itself and inflammatory cells, which represent the host immune response to the tumor. The link between lymphocyte infiltration in primary breast cancers and patient prognosis is well established in the literature [14, 26–28]. In our study, it was observed that TNBCs with a MS correlated with high TILs and showed good prognosis in both PFS and OS (Figs. 4 and 5). Ahn et al. classified breast cancer into three predominant TAS types [11]. Consistent with our findings, they observed that breast cancer cases with lymphocytic infiltration showed a favorable prognosis. Additionally, while there are many TNBC cases with high TIL, our study confirmed the remarkable presence of a low TILs in the IS (Table 2). The precise mechanism by which tumors inhibit the movement of lymphocytes remains unclear. However, there are reports that can explain this mechanism. Lieubeau et al. proposed that myofibroblasts might prevent the infiltration of immune cells into tumors, creating a physical barrier that inhibits immune reactions while promoting tumor growth and progression [29]. Our findings align with their observations, particularly the elevated ratio of low TILs observed in IS, where extracellular myxoid matrix were widely distributed, in contrast to MS. These outcomes suggest that the IS in cancer may act as a barrier, hindering the infiltration of immune cells into the tumors and promoting the survival of cancerous cells [30]. This could be one of the important reasons for the poor prognosis observed in IS with low TILs in our TNBC cases. Categorizing TAS characteristics could become a crucial indicator, helping to effectively identify patients with a favorable prognosis. Given the intricate nature of cancer progression in TNBC, further research is imperative not only to validate these tumor-specific characteristics but also to explore the diverse types of TAS and inflammatory cells involved.

## Conclusion

Our findings suggest that TAS characteristics, particularly the distinction between IS and MS, play a significant role in the prognosis of TNBC. The presence of IS, associated with poor prognosis and low TILs, contrasts with the favorable outcomes observed in cases with MS. Understanding these TAS dynamics could aid in identifying patients with varying prognostic outcomes in TNBC, necessitating further research into the mechanisms behind these observations.

## Data Availability

The datasets during the current study are available from the corresponding author on reasonable request.
